# Proteomic screen in the simple metazoan *Hydra *identifies 14-3-3 binding proteins implicated in cellular metabolism, cytoskeletal organisation and Ca^2+ ^signalling

**DOI:** 10.1186/1471-2121-8-31

**Published:** 2007-07-25

**Authors:** Barbara Pauly, Margherita Lasi, Carol MacKintosh, Nick Morrice, Axel Imhof, Jörg Regula, Stephen Rudd, Charles N David, Angelika Böttger

**Affiliations:** 1 Ludwig-Maximilians-University Munich, Germany; 2 Department of Biochemistry, Dundee University, Dundee, UK; 3 Joint Bioinformatics Laboratory, Turku Centre for Biotechnology, Turku, Finland; 4 Department of Molecular and Cell Biology, University of California, Berkeley, USA

## Abstract

**Background:**

14-3-3 proteins have been implicated in many signalling mechanisms due to their interaction with Ser/Thr phosphorylated target proteins. They are evolutionarily well conserved in eukaryotic organisms from single celled protozoans and unicellular algae to plants and humans. A diverse array of target proteins has been found in higher plants and in human cell lines including proteins involved in cellular metabolism, apoptosis, cytoskeletal organisation, secretion and Ca^2+ ^signalling.

**Results:**

We found that the simple metazoan *Hydra *has four 14-3-3 isoforms. In order to investigate whether the diversity of 14-3-3 target proteins is also conserved over the whole animal kingdom we isolated 14-3-3 binding proteins from *Hydra vulgaris *using a 14-3-3-affinity column. We identified 23 proteins that covered most of the above-mentioned groups. We also isolated several novel 14-3-3 binding proteins and the *Hydra *specific secreted fascin-domain-containing protein PPOD. In addition, we demonstrated that one of the 14-3-3 isoforms, 14-3-3 HyA, interacts with one *Hydra*-Bcl-2 like protein in vitro.

**Conclusion:**

Our results indicate that 14-3-3 proteins have been ubiquitous signalling components since the start of metazoan evolution. We also discuss the possibility that they are involved in the regulation of cell numbers in response to food supply in *Hydra*.

## Background

Apoptotic and cell survival signalling are important to maintain cellular homeostasis in many tissues and organs. This has been demonstrated very clearly during neurogenesis and haematopoesis in higher animals but has also been found at the base of metazoan evolution in the simple cnidarian *Hydra*. In *Hydra*, apoptosis is activated in response to altered feeding conditions. In well-fed animals, asexual buds are produced rapidly and cell numbers double every 2–3 days. Under restrictive feeding conditions budding stops and cell numbers do not increase. Nevertheless, cell proliferation continues, leading to the production of excess cells which are removed by apoptosis and phagocytosis [1]. Thus *Hydra *regulates organismic growth by regulating apoptosis.

In an attempt to identify molecular mechanisms, which govern the regulation of this highly useful adaptive response to feeding, we initiated a study of 14-3-3 proteins. 14-3-3 proteins are small, dimeric adaptor proteins that bind to a variety of target proteins, thereby regulating their activity, conformation, subcellular distribution and/or stability. In most cases, a 14-3-3 dimer binds to a target protein via a conserved binding motif [2,3]. These motives usually contain serine or threonine residues that become phosphorylated in response to cellular signals (recently reviewed by [4,5]). Besides these phosphorylation-dependent interactions there are a number of target proteins that bind to 14-3-3 independent of their phosphorylation state [6-8]. There is also a growing list of 14-3-3 targets which lack a conserved binding motif [8-10].

14-3-3 proteins have been implicated in the regulation of diverse cellular processes. Two important themes, however, are the involvement of 14-3-3 proteins in the regulation of metabolic responses (e.g. changes in nutrient supply) and in the regulation of apoptosis. In plants 14-3-3 proteins mediate the response of metabolic enzymes to environmental changes, e.g. sudden darkness (reviewed in [5]). The so-called "dark-induced" signalling pathway involves changes in the activity of several enzymes mediated by 14-3-3 binding after phosphorylation. In yeast, 14-3-3 proteins function in rapamycin sensitive signalling cascades as positive regulators of TOR kinase signalling [11-14]. In vertebrates the activity of key enzymes in glucose metabolism such as GAPDH and phosphofructokinase is regulated by phosphorylation through PKB/Akt and subsequent binding to 14-3-3. The involvement of 14-3-3 target proteins in apoptosis is also mediated by phosphorylation of target proteins by PKB/Akt, leading to suppression of apoptosis in the presence of growth factor signalling. The proapoptotic Bcl-2 family members Bax and Bad are examples of 14-3-3 targets in this pathway [15-17]. In the presence of growth factors Bad is phosphorylated and inactivated by binding to 14-3-3.

We have previously isolated two 14-3-3 proteins from *Hydra*, HyA and HyB, and shown that they interact with phosphorylated target proteins and form homo- and heterodimers [18]. Moreover, they respond to starvation by changing their subcellular distribution [18]. Using EST-sequences and the recently assembled *Hydra *genome we have now identified two further 14-3-3 isoforms, 14-3-3 HyC and 14-3-3 HyD, bringing the final number of 14-3-3 proteins in *Hydra *to four. To investigate the role of 14-3-3 proteins in the adaptation of hydra growth (budding) to feeding conditions we have looked for 14-3-3 target proteins. Using a 14-3-3 affinity column we identified 23 14-3-3 binding proteins in hydra extracts. Among those were a number of metabolic enzymes, cytoskeletal proteins, putative signalling molecules and novel proteins. Although Bcl-2 family members were not among the proteins isolated on the affinity column, in the Hydra EST database and in genome sequencing data at NCBI we have identified seven Bcl-2-like proteins and two Bak homologs. One of the Bcl-2-like proteins has a 14-3-3 binding motif in its C-terminus. With GST-pulldowns we show here that this *Hydra *Bcl-2-like protein interacts specifically with 14-3-3 HyA in vitro.

## Methods

### Hydra culture

*Hydra vulgaris *was cultured at a temperature of 18°C in medium containing 0.1 mM KCl, 1 mM NaCl, 0.1 mM MgSO_4_, 1 mM Tris, and 1 mM CaCl_2_. The animals were fed regularly with freshly hatched *Artemia nauplii*.

### Antibodies and reagents

Anti-14-3-3 antibody K19 was purchased from Santa Cruz Biotechnology (Santa Cruz) and used at 1:500 to 1:1000. Anti-Tubulin antibody (WA 3, used at 1:10) was a kind gift from Prof. Manfred Schliwa, Munich. Rhodamine-phalloidin was a kind gift from Dr. Ralph Gräf, Munich. Anti-mouse-FITC secondary antibody (used at 1:50) was from Sigma-Aldrich (Hamburg), anti-rabbit-Cy3 secondary antibody (used at 1:500) was from Dianova (Hamburg). Anti-digoxigenin-HRP antibody was purchased from Roche Diagnostics (Mannheim, used 1:1000) and anti-Xpress from Invitrogen (used 1:5000).

### Preparation of hydra lysate

Prior to lysis the animals were starved for two days. On the day of lysis they were washed twice in hydra medium, which was then replaced by 400 ml lysis buffer (1% Triton X-100, 1% CHAPS, 2 mM Mg-ATP, 10 μg/ml antipain/leupeptin/pepstatin A/aprotinin, 1 mM pefabloc, 1 mM vanadate, phosphatase inhibitor cocktail (Roche)). Animals were lysed by passing them through a 17 gauge needle and subsequent freezing at -80°C. The lysate was then clarified by centrifugation for 30 min at 30,000 g and 4°C and the supernatant was mixed with 8 mg Bmh1/Bmh2-CH-sepharose 4B for 1 h at 4°C.

### 14-3-3 affinity column

The 14-3-3 affinity column was prepared essentially as described in Moorhead et al [19]. Briefly, 8 mg recombinant 14-3-3 from *Saccharomyces cerevisiae *(Bmh1 and Bmh2, 6× His tagged, expressed in E. coli DH5α, purified as in [19,20]), was incubated with activated CH sepharose at room temperature. Non-reacted, active groups were blocked with 0.1 M Tris/Cl pH 8. A lysate prepared from 100 000 hydra was incubated with 14-3-3 sepharose for 1 h at 4°C. The mixture was then packed into a disposable plastic column (Biorad) and washed with 50 mM HEPES, pH 7.5/0.5 M NaCl/1 mM DTT. To test whether proteins were eluted unspecifically, the column was washed with an unrelated phospho-peptide (-WFYpSFLE-). The 14-3-3 binding proteins were then eluted from the column with 1 mM peptide C (-ARAApSAPA-). The presence of 14-3-3 binding proteins in every fraction was tested in a Far Western Overlay with DIG labelled 14-3-3 proteins.

### Mass Spectrometry and EST analysis

20 μg of eluted 14-3-3 binding proteins were separated in an SDS-gel and stained with colloidal coomassie. Single protein bands were cut out of the gel and washed twice with water and twice with 40 mM ammoniumbicarbonate. After two-fold treatment with 50% acetonitrile for 5 min, 10 μg/ml trypsin (Promega) was added and proteins were digested overnight in 40 mM ammoniumbicarbonate at 30°C while shaking. For protein identification probes were directly used for nano-ESI-LC-MS/MS. Each sample was first separated on a C18 reversed phase column via an acetonitrile gradient (Famos-Switchos-Ultimate System and column (75 μm i.d. × 15 cm, packed with C18 PepMap™, 3 μm, 100 Å) by LC Packings) before spectra were recorded on a QSTAR XL mass spectrometer (Applied Biosystems). The resulting spectra where then analysed via the Mascot™ Software (Matrix Science) using the *Hydra *Protein database (see below).

### Peptide prediction from Hydra EST sequences

*Hydra *EST sequences were downloaded from the EMBL sequence database [21] and were assembled on the Sputnik comparative genomics platform [22]. These sequences are derived from several cDNA libraries made from whole budding hydra (*Hydra *EST database). To increase the relative quality of the sequences and to reduce sequence redundancy sequence clustering was performed using the HarvESTer application.

All unigene sequences were compared against known or predicted peptides using the BLASTX algorithm against a non-redundant (Nonred) protein sequence database. Best scoring BLASTX matches exceeding the arbitrary expectation value of 10e^-10 ^were selected and the *Hydra *coding sequence was extracted from the BLAST output. 1,853 high scoring sequence blocks were selected. These sequence blocks were scored for the relative occurrence of all in-frame hexanucleotide sequences. The concomitant di-codon probability tables were used with the frame finder application to select for the most parsimonious open reading frame from each unigene sequence. This yielded robust peptide sequences even in the absence of a BLASTX homologue.

### DIG labelling of 14-3-3 proteins

Recombinant 14-3-3 from yeast (Bmh1 and Bmh2, see above) was labelled with digoxigenin-3-O-methylcarbonyl-e-aminocaproic acid-N-hydroxysuccinimide ester and separated from excess reagent using the digoxygenin protein labeling kit (Roche Diagnostics) according to the manufacturer's protocol. Labelled 14-3-3 protein was diluted to a final concentration of 1 μg/ml in 2 mg/ml BSA and 0.05% sodium azide and stored at 4°C.

### Far Western Overlay

The overlay assays were carried out as described by Moorhead et al. [20]. In brief, after SDS-PAGE and Western blotting blots were probed with DIG labelled 14-3-3 proteins and HRP-labelled anti-DIG antibody (Roche Diagnostics) according to the manufacturer's instructions. ECL was used for detection.

### Immunoprecipitation

For immunoprecipitation experiments, hydra cellular lysates were made in lysis buffer (1% Triton X-100, 1% Chaps, phosphatase- and protease inhibitors). Lysates were incubated with 5 μg of the anti-14-3-3 antibody K19 (Santa Cruz) for 1 h at 4°C. Protein A (Amersham Biosciences) was added and the samples were incubated for an additional hour at 4°C. Samples were then spun down and pellets were washed three times with lysis buffer and one time with Tris buffer. After addition of SDS loading buffer, the proteins were separated by SDS PAGE and subjected to Western blot analysis. In cases when peptide C was used, hydra lysates were incubated with 1 mM peptide C for 2 h at 4°C prior to immunoprecipitation. In samples treated with phosphatase, the lysates were incubated with 200 U λ-phosphatase (New England Biolabs) for 30 min at 30°C prior to immunoprecipitation.

### Expression of GFP fusion proteins in Hydra

To transiently express GFP-fusion proteins in hydra, the corresponding genes were introduced into hydra using the PDS-1000/He Particle Delivery System (Biorad) as described in [23]. Briefly, 20 μg of DNA was added to 3 mg of gold particles (1 μm diameter) and precipitated with 0.3 M sodium acetate and 2.5 vol ethanol. Coated particles were washed with 70% ethanol, resuspended in 200 μl ethanol, and spread onto carrier disks according to the manufacturer's instructions. Hydra were collected in petri dishes and as much medium as possible was withdrawn. The animals were then shot 3 times with the gold particles. 2–3 days after transformation animals were screened for expression of the GFP fusion protein.

### Immunofluorescence

For immunofluorescence animals were relaxed in 2% urethane for 2 min and then fixed for 1 h at room temperature with Lavdovsky (formaldehyde : acetic acid : ethanol : water 5:2:25:20). After fixation animals were permeabilised in 0.5% Triton X-100 and unspecific binding sites were blocked with 1% BSA/0.1% Triton X-100. Incubation with primary antibody was carried out over night at 4°C in blocking solution, followed by washes with phosphate buffered saline and incubation with fluorescently labelled secondary antibody for 2 h at room temperature. Animals were counterstained with TO-PRO3 (Molecular Probes) and mounted in Vectashield (Vector Laboratories) to prevent bleaching. Animals were analysed by confocal microscopy.

### Confocal microscopy

Light optical serial sections were acquired with a Leica (Leica Microsystems, Heidelberg) TCS SP confocal laser scanning microscope equipped with an oil immersion Plan-Apochromat 100/1.4 NA objective lens. Fluorochromes were visualised with an argon laser with excitation wavelengths of 488 nm, emission filter 520–540 nm (for FITC) and with a helium-neon laser with excitation wavelength of 633 nm, and emission filter 660–760 nm (for TO-PRO3 and TRITC). Two fluorochromes and the phase contrast image (transmission filter) were scanned sequentially. Image resolution was 512 × 512 pixel with a pixel size ranging from 195 to 49 nm depending on the selected zoom factor. The axial distance between optical sections was 300–500 nm for zoom factor 4 and 1 μm for zoom factor 1. To obtain an improved signal-to-noise ratio each section image was averaged from four successive scans. The 8-bit greyscale single channel images were overlayed to an RGB image assigning a false colour to each channel, and then assembled into tables using ImageJ 1.32j and Adobe PhotoShop 5.5 software.

### GST-pulldown

14-3-3HyA and 14-3-3HyB were cloned into the plasmid pRSET, expressed in bacteria and purified as described previously [18]. Hybcl-2-like1 was cloned into the vector pGEX and expression of GST-Hybcl-2-like1 or GST-Hyinnexin 1 as a control was induced. Equal amounts of bacterial lysates were incubated with glutathione-sepharose beads for 2 h at room temperature under constant agitation. The beads were sedimented by centrifugation, washed and subsequently incubated with the purified *Hydra *14-3-3 proteins for 2 h at room temperature under constant agitation. After centrifugation and washing with PBS, bound proteins were eluted with reduced glutathione and subjected to SDS-PAGE and immunoblotting with anti-Xpress (for proteins expressed from pRSET) and anti-GST antibodies (27457701, GE Healthcare).

## Results

### Hydra has four 14-3-3 isoforms

**Figure 1:**
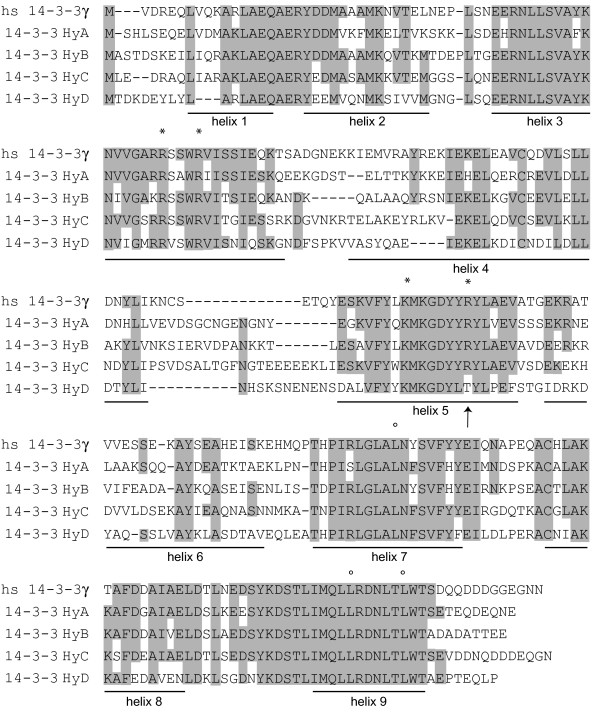
**Alignment of human 14-3-3 with four isoforms of Hydra 14-3-3**. Full length clones of 14-3-3 HyA and 14-3-3 HyB isoforms were described previously [18]. 14-3-3 HyC and 14-3-3 HyD were identified in expressed sequence tags from *Hydra *at NCBI. Helices 1-9 are indicated. Conserved amino acids that have been shown in the crystal structure to constitute the binding groove for target proteins are indicated with asterisks (basic face of the groove) and circles (hydrophobic surface) above the sequence (information from [24]). Alignments were made with the program GeneJockeyII (under MacOS9)

Two 14-3-3 isoforms, HyA and HyB, have previously been isolated from *Hydra *by RT-PCR [18]. By searching the now available *Hydra *EST database (ca. 170 000 sequences) we have identified two additional 14-3-3 isoforms, which we named 14-3-3 HyC and 14-3-3 HyD. Alignment of all four 14-3-3 proteins from *Hydra *with human 14-3-3 γ shows that the basic features of 14-3-3 proteins are well conserved in the *Hydra *sequences including nine α-helices and a number of amino acids lining the amphipathic groove which constitutes the binding site for 14-3-3 target proteins [24]. In Fig. 1 amino acids of the hydrophobic surface of the groove are shown with circles above the alignment. These are completely conserved in all four 14-3-3 isoforms from *Hydra*. Amino acids on the basic face of the groove are indicated with asterisks. They are also conserved except for the arginine in helix 5, which is replaced by threonine in 14-3-3 HyD. This would probably have implications for the binding of target proteins to this isoform.

**Figure 2:**
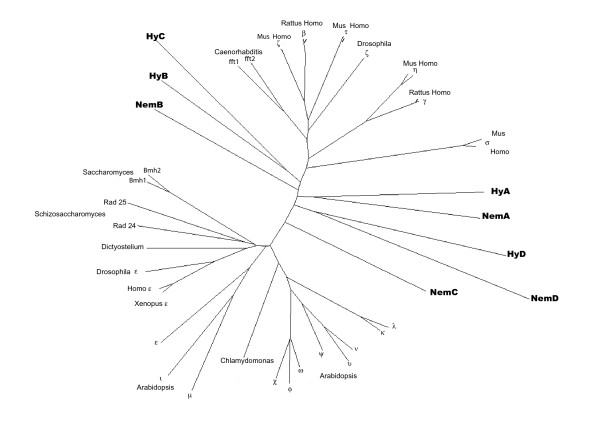
**Unrooted phylogenetic tree**. Tree includes 14-3-3 isoforms from plant and animal species and four isoforms from *Hydra *and from *Nematostella*. The cnidarian isoforms are printed in bold, Nem = *Nematostella*, Hy = *Hydra*.

We have also identified four 14-3-3 isoforms in the recently completed genome of another cnidarian, the sea anemone *Nematostella vectensis*. We have named these proteins 14-3-3 NemA, 14-3-3 NemB, 14-3-3 NemC and 14-3-3 NemD with reference to the *Hydra *isoforms. A phylogenetic tree including representatives of all known plant and animal isoforms as well as sequences from *Chlamydomonas, Dictyostelium, Saccharomyces and Schizosaccharomyces *in addition to the cnidarian sequences is presented in Fig. 2. It shows that the eight cnidarian 14-3-3 proteins do not fall into the established isoform groups. Among themselves, however, they seem to form three groups, which separated very early. This correlates with the ancient separation of anthozoans and hydrozoans. The number and diversity of *Hydra *and *Nematostella *14-3-3 proteins distinguishes the 14-3-3 repertoires in cnidarians from them in other invertebrates. *Drosophila *and *C. elegans *each have only two 14-3-3 proteins and these are grouped with metazoan isoforms [18].

### Isolation of 14-3-3 binding proteins from hydra lysates

**Figure 3:**
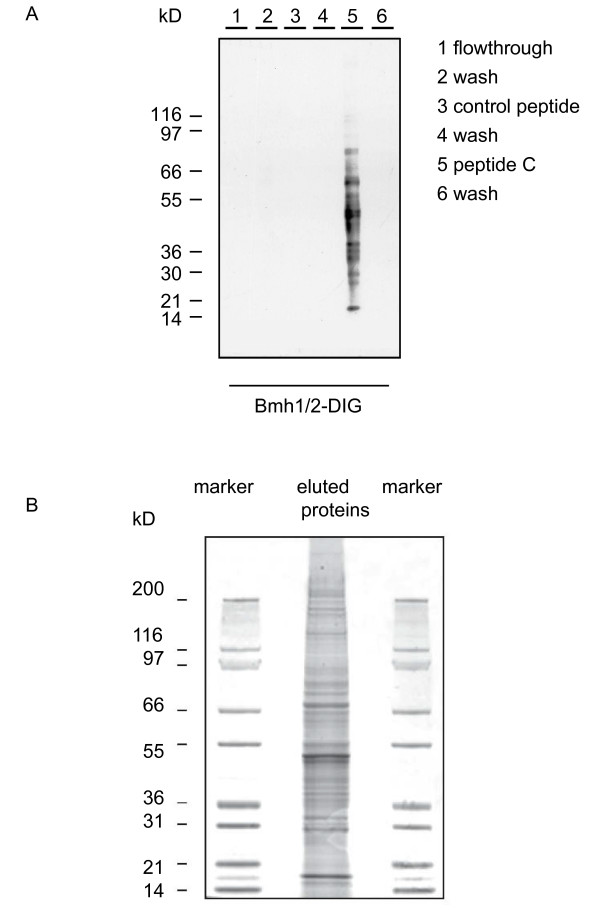
**Fractions after elution from 14-3-3 affinity column**. (A) SDS PAGE gel of fractions eluted from Bmh1/2-affinity column, Western blot probed with DIG labelled Bmh1/2 from yeast. 14-3-3 binding proteins are only in the fraction that was eluted with 14-3-3 consensus peptide C (lane 5). (B) SDS-PAGE gel stained with coomassie; lanes 1 and 3 show size markers as indicated.

In Far Western overlays with DIG labelled 14-3-3 probes we have previously shown the presence of a large number of 14-3-3 binding proteins in hydra extracts. Binding to 14-3-3 was dependent on the phosphorylation of these targets and it involved the conserved binding pocket of 14-3-3 proteins because it could be blocked with the phosphorylated 14-3-3 consensus peptide ARAApSAPA [18]. To establish the molecular identity of these target proteins we isolated them using a 14-3-3-affinity column. Extracts were prepared from ca. 100,000 hydra. These were incubated with 14-3-3 (Bmh1/2 from yeast) coupled sepharose and the mixture was packed into a column (described in [19]). The column was washed extensively with 50 mM HEPES, 0.5 MNaCl, 1 mM DTT, and then with an unrelated phosphopeptide (-WFYpSFLE-). Elution of 14-3-3 binding proteins was carried out with 1 mM of phosphorylated peptide C (-ARAApSAPA-) which constitutes a consensus binding site for 14-3-3 proteins and thus competes with target proteins for binding to 14-3-3 [3,24]. The eluted proteins were concentrated, separated in SDS-PAGE and probed with DIG labelled 14-3-3. Fig. 3A shows the presence of about 20 14-3-3-binding proteins specifically enriched in the final eluate (lane 5). To analyse these proteins they were stained with coomassie (Fig. 3B). Visible bands were then cut out, the proteins eluted and digested with trypsin. For protein identification, probes were directly used for nano-ESI-LC-MS/MS. The resulting spectra were analyzed with the Mascot™ Software (Matrix Science) using a *Hydra *protein database. *Hydra *peptides generated from the EST data (see Materials and Methods) were trypsinised *in silico*. The masses of the tryptic peptides were calculated and compared with the MALDI mass data of the isolated 14-3-3 binding proteins. This allowed identification of 23 proteins. These are shown in Table 1. The identity of the isolated proteins is given in column 1. Column 2 indicates the overall LC-MS score and the number of peptides for which sequencing data were obtained. We then compared the molecular weights of the identified proteins with their sizes deduced from their position in SDS-PAGE. In two cases where the EST sequence did not encode a full-length cDNA, the sizes of the human (hs) or *Drosophila *(dm) homologues are indicated. All identified proteins corresponded in size with the expected molecular weight of their bands in SDS-PAGE. Moreover, 13 of the proteins had 14-3-3 binding motifs, 11 had been previously reported in the literature to bind to 14-3-3 in plant or animal cells [25-32]. Two of the previously unknown 14-3-3 binding partners are proteins that have so far been exclusively found in *Hydra*.

**Table 1:** List of proteins purified from hydra lysates by 14-3-3-affinity chromatography

**identification**	**protein score (matched peptides)**	**size of hydra protein (kDa)**	**size of gel band (kDa)**	**14-3-3 binding motif**	**previously identified 14-3-3 target**
**cytoskeleton**					
tubulin α	102 (6)	53	53	no	[32]
tubulin β	89 (5)	50	50	yes	[32]
actin	186 (8)	41	40	yes	[27, 32]
tropomyosin	101 (7)	27	37	yes	[32]
**calcium signalling**					
calmodulin	71 (6)	17	17	no	[30, 32]
calcium binding prot.	134 (9)	20	20	no	
calcium adaptor AIF-1	112 (7)	17	17	no	
**metabolism**					
fru-1,6-BP-aldolase	98 (6)	36	41	no	
GAPDH	172 (9)	36.5	37	yes (3)	[26, 28, 32]
PEPCK	53 (3)	71 (hs)	64	yes (hs)	
ATP-synthase β	217 (13)	56	50	yes (2)	[25, 32]
**protein biosynthesis/-folding**					
EF-2	51 (3)	97	96	yes	[32]
EF1 α	156 (8)	51	50	yes (2)	[32]
eIF-3 β	132 (5)	36	36	yes (2)	
Hsp70	174 (10)	75	70	yes	[29, 31, 32]
PDI-like protein	78 (6)	48 (dm)	50	yes	
**Miscellaneous**					
ppod3	212 (10)	30	30	no	
ppod4	290 (8)	32	32	yes	
14-3-3 HyA	157 (6)	28.6	30	-	
14-3-3 HyB	187 (8)	28.1	30	-	
unknown protein	51 (4)	19	32	no	
hypothetical protein	95 (6)	24	80	no	

Protein scores and numbers of identified peptides from analysis of mass spectra using Mascot™ Software are given in column 2, columns 3 and 4 compare the molecular weights of the identified proteins with the apparent molecular weights of these proteins on the SDS-PAGE gel from which they had been cut out for analysis. In column 5 the presence of a conserved 14-3-3 binding motif in the sequence of the identified proteins is indicated [2, 3]. Column 6 lists citations that have described interactions of 14-3-3 proteins with plant or animal homologues of the identified proteins before. Abbreviations: dm: *Drosophila melanogaster*; hs: *Homo sapiens*

### Identified 14-3-3 binding proteins

#### Cytoskeletal proteins

**Figure 4:**
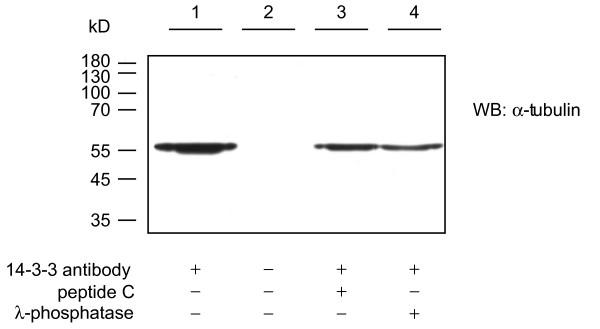
**Immunoprecipitation of tubulin from *Hydra *lysate with anti-14-3-3 antibody**. Western blot stained with anti-tubulin antibody. Lane 1 shows tubulin precipitated with anti-14-3-3 antibody from hydra lysates, in lane 2 the anti-14-3-3 antibody was omitted as control for non-specific binding of proteins to the beads, in lane 3 peptide C was added before precipitation with anti-14-3-3-antibody and in lane 4 the lysate was treated with λ-phosphatase before preciptiation with anti-14-3-3 antibody.

One group of the identified *Hydra *14-3-3 binding proteins is part of the cytoskeleton (tubulin α and β, actin, tropomyosin). We have confirmed the interaction of tubulin with 14-3-3 in an immunoprecipitation experiment (Fig. 4). Interestingly, the interaction was only very weakly inhibited by peptide C or phosphatase treatment. This could suggest that the interaction is very strong and bound 14-3-3 protein shields the phosphate group from the phosphatase. Alternatively 14-3-3 might bind to tubulin indirectly or via an uncharacterised mechanism. For β-tubulin, which contains a classical 14-3-3 binding motif, this would be unexpected. It is, however, possible that the antibody used in the immunoprecipitation experiment preferentially recognises α-tubulin.

**Figure 5:**
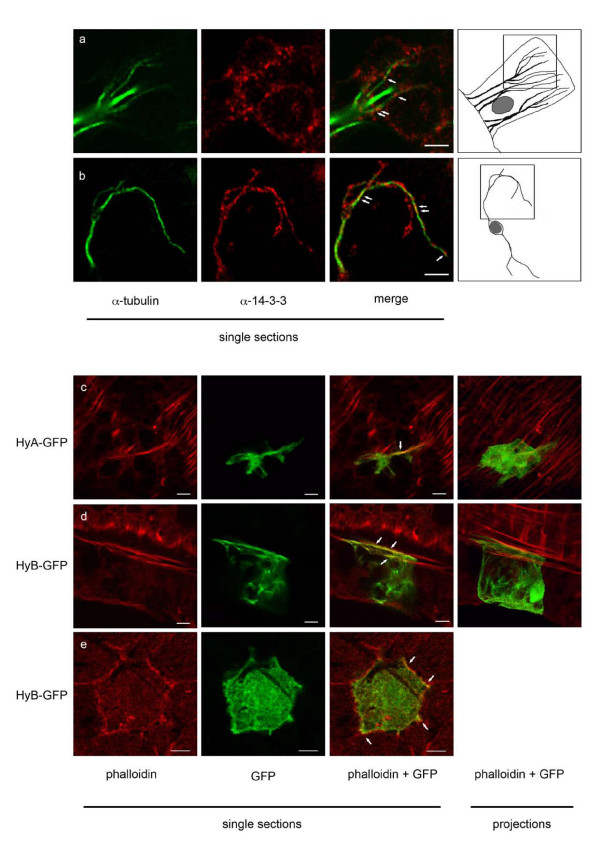
**Co-localisation of Hydra 14-3-3 proteins with tubulin and actin**. (a, b) right hand panel shows schematic drawings of an epithelial cell (a) and a nerve cell (b) in hydra macerates that were stained with anti-tubulin (green, left hand panel) and anti-14-3-3 antibodies (second panel from left). Single optical sections after laser scanning confocal microscopy from the indicated regions are shown. Merged images (third panel from left) indicate partial co-localisation of 14-3-3 with tubulin (indicated by arrows); (c-e) single confocal sections of basal parts of hydra cells expressing 14-3-3 HyA-GFP (c) or 14-3-3 HyB-GFP (d) in whole mounts stained with rhodamine-phalloidin (red); e: single section of the apical part of an epithelial cell expressing 14-3-3 HyB-GFP counterstained with rhodamine-phalloidin. In the merged images, white arrows indicate co-localisation of 14-3-3 with actin. Scale bars are 10 μm in c and d, and 5 μm in e.

In order to further confirm the interaction of 14-3-3 with components of the *Hydra *cytoskeleton we performed double immunofluorescence with anti-tubulin and anti-14-3-3 antibodies. The result is shown in Fig. 5 panels a and b. 14-3-3 shows the punctate distribution in macerated epithelial cells that we have described previously [18]. These structures are partially co-localised with microtubules (Fig. 5 panels a and b, merge, indicated by arrows) indicating that an interaction of 14-3-3 and tubulin takes place only at specific locations on microtubuli. Tubulin has been isolated as a 14-3-3 binding protein in two previous proteomic screens [32,33]. In one of those screens the same approach as used in our study had been taken with a Bmh1/2 affinity column to purify 14-3-3 binding proteins from HeLa cell extracts. The other study used HEK293 cells constitutively expressing Flag-tagged human 14-3-3 γ and binding proteins were captured by immunoprecipitation with an antibody that recognised the Flag-tag. Direct interaction between tubulin and 14-3-3 proteins has not been demonstrated so far in mammals or in *Hydra *and our experiments do not exclude the possibility that we are looking at larger protein complexes containing 14-3-3 and tubulin. Microtubule associated proteins have been shown to bind to 14-3-3 previously, e.g. tau. In this case, however, the interactions between tau and tubulin or 14-3-3 ζ were mutually exclusive [34,35].

To look for co-localisation of 14-3-3 HyA and 14-3-3 HyB with the actin cytoskeleton we used cells transiently transfected with GFP-tagged 14-3-3 proteins and counterstained them with rhodamine-phalloidin. Fig. 5, panels c and d, show sections through the basal part of 14-3-3 HyA-GFP and 14-3-3 HyB-GFP expressing ectodermal epithelial cells, respectively. Actin filaments are present in the muscle processes of these epithelial muscle cells. A small fraction of both 14-3-3-GFP isoforms was seen co-localised with phalloidin in these processes. Fig. 5, panel e, shows sections through the apical end of an ectodermal epithelial cell expressing 14-3-3 HyB-GFP. The phalloidin signal representing cortical actin co-localises especially strongly with 14-3-3 HyB-GFP at the cell boundaries (white arrows).

Interaction of actin with 14-3-3 has also been described in the past. In primary cultures of cerebral cortical astrocytes 14-3-3 γ was associated with actin filaments and this association was lost under conditions of actin reorganisation during mitosis and actin disruption in apoptotic cells [27]. By stabilising phospho-cofilin 14-3-3 plays an important role in actin filament turnover in motile cells [36]. Rearrangement of cortical actin prior to secretion of exocytic vesicles has also been shown to depend on 14-3-3 proteins [37,38]. In yeast, disruption of 14-3-3 signalling by overexpression of a dominant negative 14-3-3 variant or of a temperature sensitive 14-3-3 mutant at the restrictive temperature has dramatic effects on the organisation of the actin cytoskeleton [39,40].

#### Calcium binding proteins

Three Ca^2+ ^binding proteins (calmodulin, Ca^2+ ^binding protein and Ca^2+ ^adaptor AIF1 (allograft inflammatory factor 1)) constitute a second group of the identified hydra 14-3-3 binding proteins. Interaction of calmodulin with human 14-3-3 ε had been shown previously in human cells [30]. The consequences of this interaction are not very well known. With respect to a role for 14-3-3 proteins in Ca^2+ ^signalling pathways some examples in the literature describe interaction of 14-3-3 proteins with targets that are phosphorylated by Ca^2+^/calmodulin dependent kinase, e.g. the photoreceptor regulating protein phosducin [41] and the histone deacetylase 7 (HDAC7) [42]. Alternatively, such a 14-3-3 binding site can also be abolished by a Ca^2+^stimulated phosphatase as has been shown for Ca^2+^activated nuclear factor of activated T-cells which acts at the interleukin-2 promoter and is negatively regulated by 14-3-3 [43]. Moreover, Ca^2+^/calmodulin dependent kinase kinase (CaMKK) is directly regulated by binding to 14-3-3 after phosphorylation by PKA [44].

With the *Hydra *Ca^2+ ^binding protein and the Ca^2+ ^adaptor AIF-1 we have discovered two new 14-3-3 target proteins that are potential members of Ca^2+ ^signalling pathways. They both have an EF-hand Ca^2+ ^binding motif. The former is related to a family of flagellar Ca^2+ ^binding proteins that is highly conserved in trypanosoma [45]. The closest homolog of the *Hydra *Ca^2+ ^adaptor protein AIF-1 is the allograft inflammatory factor 1 from the sponge *Suberites domuncula *[46]. AIF-1, which was first discovered in rat cardiac allografts undergoing chronic rejection, has thus homologs in vertebrates and lower invertebrates [47]. In higher animals it plays a role in vascular remodelling and repair which probably involves induction of actin polymerisation in vascular smooth muscle cells and is dependent on an intact EF-hand motif [48,49]. The discovery of these two new 14-3-3 target proteins suggests a broader role of 14-3-3 proteins in Ca^2+ ^signalling.

#### PPOD

PPOD 3 and 4 belong to a novel protein family found only in *Hydra *[50,51]. These proteins possess fascin domains and are localised in secretory granules. They are secreted proteins as was shown in a recent genetic screen for signal peptides in *Hydra *[22]. Moreover, they are able to agglutinate erythrocytes and thus may have lectin character (Pauly 2006, unpublished observations). They represent novel 14-3-3 binding proteins. In this context it is interesting to note that 14-3-3 target proteins have been isolated from organelles like mitochondria, chloroplasts or Golgi vesicles and secreted forms of 14-3-3 proteins have also been described [32,52-55]. Their sorting however remains enigmatic since 14-3-3 proteins do not have a signal peptide for targeting them to any of these organelles.

#### Metabolic enzymes

Finally, seven of the identified 14-3-3 binding proteins are metabolic enzymes involved in glucose metabolism (glyceraldehydephosphate dehydrogenase (GAPDH, EC1.2.1.12.), phosphoenolpyruvate carboxykinase (PEPCK), fructose-1,6-biphosphate aldolase), in protein synthesis and folding (EF-2, elongation factor 1 α, eIF-3 β) or ATP synthesis (ATP-synthase, [25]). The interactions of 14-3-3 with fructose-1,6-aldolase and PEPCK have been seen for the first time in our study. In contrast, the classical glycolytic enzyme GAPDH has been shown to bind to 14-3-3 proteins in several organisms, including wheat [26], cauliflower [19] and human HeLa cells [32]. Studying the significance of the 14-3-3/GAPDH interaction is complicated by the fact that GAPDH is a multifunctional protein. In addition to its participation in carbohydrate metabolism it also plays roles in membrane fusion, microtubule bundling, nuclear RNA export, DNA repair, DNA replication, translational regulation, and apoptosis (reviewed by [56-58]). Interestingly, GAPDH is translocated to the nucleus early in apoptosis and nuclear overexpression of this enzyme potently induces cell death [59,60]. Serum withdrawal (or growth factor deprival) also leads to nuclear translocation of GAPDH. This is regulated by a PI(3)kinase dependent pathway [61]. However, at the moment we don't know anything about the significance of 14-3-3 binding to GAPDH in animal cells. In *Arabidopsis *cells it was shown that 14-3-3 stabilises metabolic enzymes when they are needed. In sugar starved cells 14-3-3 binding is lost and this leads to rapid degradation of metabolic 14-3-3 target proteins [28]. Such a clear picture does not exist for any of the metabolic 14-3-3 target proteins that have recently been found in large screens in mammalian cells [32,33]. Nevertheless, the identification of 14-3-3 binding enzymes that are directly involved in carbohydrate metabolism in *Hydra *together with the previously described redistribution of 14-3-3 proteins in starving animals [18] strengthen our hypothesis that growth regulation in response to nutrition in *Hydra *could involve 14-3-3 proteins.

### 14-3-3 HyA interacts with one Hydra Bcl-2 family member

**Figure 6:**
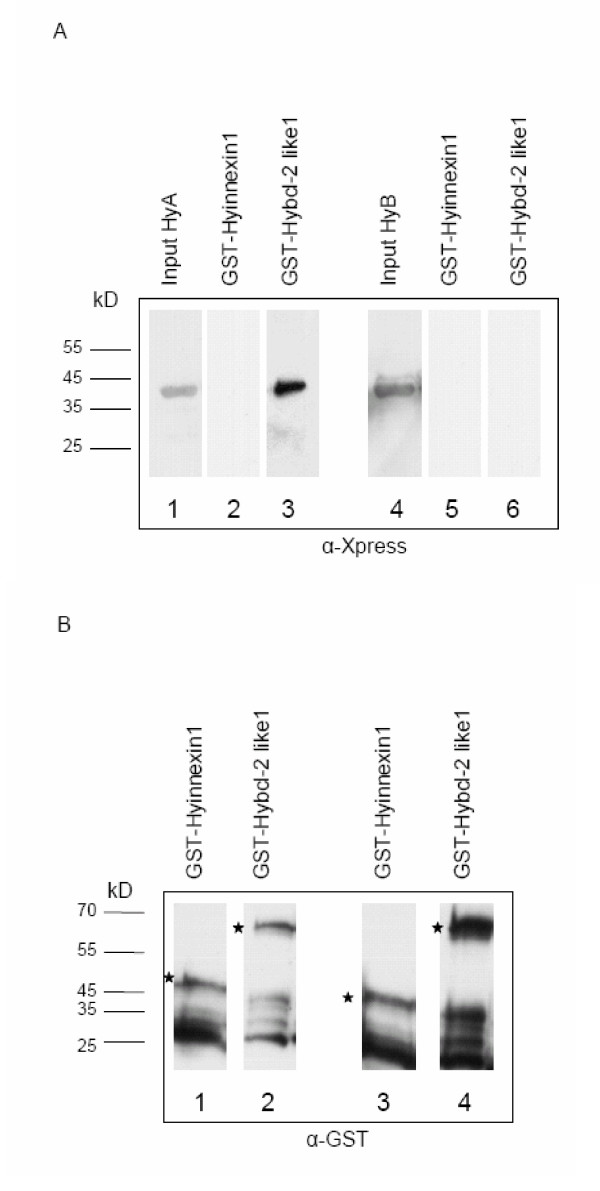
**14-3-3 HyA interacts with *Hydra *Bcl-2-like protein in vitro**. (A) bacterially expressed His-tagged 14-3-3 HyA or 14-3-3 HyB were incubated with bacterial lysates containing GST-Hybcl-2-like1 or as a control GST-HyInnexin1 (lanes 1-6). Inputs of 14-3-3 HyA (lane 1) and 14-3-3 HyB (lane 4) represent 12.5% of the total 14-3-3 proteins used in each assay. Eluted 14-3-3 HyA (lanes 2 and 3) and 14-3-3 HyB (lanes 5 and 6) are shown after pulldown with GST-fusion-proteins. Western blots were probed with anti-Xpress antibody, which recognises the His-tag of the bacterially expressed 14-3-3 proteins. In (B) Western blots with samples from (A) were probed with anti-GST antibody to show that equal amounts of GST-fusion-protein were eluted in all assays. Asterisks indicate full-length GST-fusion proteins. Smaller fragments represent degradation products.

Several members of the Bcl-2 family of apoptotic regulators are known to bind to 14-3-3, e.g. Bax and the BH-3 only protein Bad. Hence we had anticipated finding these among proteins eluted from the 14-3-3 affinity column. Our failure to do so could have been due to low abundance of these proteins in the extracts. We searched the *Hydra *EST database to confirm the presence of Bcl-2 family members in *Hydra *and identified seven *bcl-2 like sequences *and two *bak *homologues (Lasi and Pauly, manuscript in preparation) but no BH-3 only proteins like Bad. For three of these proteins we obtained complete cDNAs by RT-PCR (not shown). These have been submitted to GenBank, accession numbers are: *Hybcl-2-like1 *(EF104645), *Hybak-like *(EF104646), *Hybcl-2-like2 *(EF104647). HyBcl-2-like1 contained the sequence RTFTKP, a conserved 14-3-3-binding motif [3], in its C-terminus. Work in vertebrate cells has shown that the interaction of Bax with 14-3-3 proteins is not dependent on phosphorylation of Bax [15,62]. If this was true for the hydra protein it might not have been expected to be eluted from the 14-3-3 affinity column with the phosphorylated peptide C. In order to test the possibility that Hybcl-2-like1 could be a target for 14-3-3 we carried out a GST pulldown assay with bacterially expressed 14-3-3 HyA and 14-3-3 HyB. GST-Hybcl-2-like1 was incubated with purified N-terminally His-tagged 14-3-3 HyA and 14-3-3 HyB, respectively. As shown in Fig. 6 14-3-3 HyA could be pulled down with GST-Hybcl-2-like1 (Fig. 6A). In contrast, neither 14-3-3 HyB nor Hyinnexin (control) were precipitated. The presence of equal amounts of the GST fusion proteins in all eluates was confirmed by probing the samples with anti-GST antibody (Fig. 6B). Thus, 14-3-3 HyA specifically interacts with Hybcl-2-like1. This specificity may have been another reason why we did not find this protein in the affinity purification experiment, which had been carried out with yeast 14-3-3 proteins. However, the GST-pulldown indicates that 14-3-3 proteins in *Hydra *also have potential targets in the Bcl-2 family of pro- and anti-apoptotic proteins.

## Conclusion

In a proteomic screen we have identified 23 14-3-3 binding proteins in the early metazoan *Hydra*. They fall into several groups, including cytoskeletal proteins, proteins implicated in Ca^2+ ^signalling and, interestingly, proteins involved in protein synthesis and metabolism. Moreover, we showed that one 14-3-3 *Hydra *isoform can interact with the Bcl-2 family member Hybcl-2-like1 in vitro. The functional implications of 14-3-3 binding to these metabolic and apoptotic target proteins have not been investigated yet. It is possible that some of these proteins are phosphorylated by PKB. PKB has been identified in *Hydra *[63]. Moreover, hydra cells are sensitive to inactivation of PKB signalling with the PI(3) kinase inhibitor wortmannin, which induces massive apoptosis [64]. This raises the possibility that survival factor signalling is involved in the adaptation of hydra growth to food supply and that 14-3-3 proteins mediate this response both on the level of metabolic control and on the level of apoptosis induction.

On a wider perspective our study has made it clear that 14-3-3 targets in the early metazoan *Hydra *are as diverse as in higher animals, including humans. Our screen only allowed detection of the most abundant target proteins and yet they contained members of groups of proteins with diverse functions such as metabolism, cytoskeletal regulation, Ca^2+^-signalling and apoptosis. It thus appears that the ubiquitous presence of 14-3-3-proteins in all eukaryotic organisms studied so far is accompanied by a wide diversity of target proteins even in the simplest metazoans.

## Competing interests

The author(s) declare that they have no competing interests.

## Authors' contributions

BP carried out the 14-3-3 affinity column screen and interaction studies with cytoskeletal proteins, ML performed GST pull-downs with Bcl-2 family proteins. CM participated in designing and performing the screen, NM, AI and JR performed mass spectromery, SR provided the peptide prediction from Hydra EST data, CND participated in the design of the study, AB designed and coordinated the study and drafted the manuscript.
